# Seroprevalence of Antibodies against SARS-CoV-2 among the Personnel and Students of the National and Kapodistrian University of Athens, Greece: A Preliminary Report

**DOI:** 10.3390/life10090214

**Published:** 2020-09-21

**Authors:** Ourania E. Tsitsilonis, Dimitrios Paraskevis, Evi Lianidou, Vassilios Pierros, Athanasios Akalestos, Efstathios Kastritis, Paraskevi Moutsatsou, Andreas Scorilas, Thomas Sphicopoulos, Evangelos Terpos, Nikolaos Thomaidis, Athanassios Tsakris, Nikolaos Voulgaris, Christina C. Daskalaki, Zoi Evangelakou, Christina Fouki, Despoina D. Gianniou, Sentiljana Gumeni, Evangelia-Georgia Kostaki, Ioannis V. Kostopoulos, Maria S. Manola, Nikolaos Orologas-Stavrou, Chrysanthi Panteli, Eleni-Dimitra Papanagnou, Pantelis Rousakis, Aimilia D. Sklirou, Stavroula Smilkou, Dimitra Stergiopoulou, Ioannis P. Trougakos, Soritios Tsiodras, Petros P. Sfikakis, Meletios-Athanasios Dimopoulos

**Affiliations:** 1 Department of Biology, National and Kapodistrian University of Athens (NKUA), 15784 Athens, Greece; rtsitsil@biol.uoa.gr (O.E.T.); ascorilas@biol.uoa.gr (A.S.); xristin1.dask@gmail.com (C.C.D.); zoievag@biol.uoa.gr (Z.E.); gndespoina@biol.uoa.gr (D.D.G.); sgumeni@biol.uoa.gr (S.G.); gikosto@gmail.com (I.V.K.); mmanola@biol.uoa.gr (M.S.M.); norologas@biol.uoa.gr (N.O.-S.); chrysanthipanteli23@gmail.com (C.P.); epapanagnou@biol.uoa.gr (E.-D.P.); rousakisp@gmail.com (P.R.); asklirou@biol.uoa.gr (A.D.S.); itrougakos@biol.uoa.gr (I.P.T.); 2 Department of Hygiene Epidemiology and Medical Statistics, School of Medicine, NKUA, 15772 Athens, Greece; dparask@med.uoa.gr (D.P.); chrfouki@hotmail.com (C.F.); ekostakh@med.uoa.gr (E.-G.K.); 3 Department of Chemistry, NKUA, Athens 15771, Greece; lianidou@chem.uoa.gr (E.L.); ntho@chem.uoa.gr (N.T.); ssmilkou@chem.uoa.gr (S.S.); dimitrastergiopoulou@yahoo.com (D.S.); 4 Department of Informatics and Telecommunications, NKUA, 15784 Athens, Greece; pierrosv@di.uoa.gr (V.P.); thomas@di.uoa.gr (T.S.); 5 Roche Diagnostics (Hellas) S.A., 15125 Marousi, Greece; thanasis.akalestos@roche.com; 6 Department of Clinical Therapeutics, School of Medicine, Alexandra General Hospital, NKUA, 11528 Athens, Greece; ekastritis@med.uoa.gr (E.K.); eterpos@med.uoa.gr (E.T.); 7 Department of Clinical Biochemistry, School of Medicine, University General Hospital Attikon, NKUA, 12462 Haidari, Greece; pmoutsatsou@med.uoa.gr; 8 Department of Microbiology, School of Medicine, NKUA, 11527 Athens, Greece; atsakris@med.uoa.gr; 9 Department of Geology and Geoenvironment, NKUA, 15784 Athens, Greece; voulgaris@geol.uoa.gr; 10 Fourth Department of Propedeutic Internal Medicine, School of Medicine, University General Hospital Attikon, NKUA, 12462 Haidari, Greece; tsiodras@med.uoa.gr; 11 First Department of Propaedeutic Internal Medicine, School of Medicine, Laiko General Hospital, NKUA, 15772 Athens, Greece; psfikakis@med.uoa.gr

**Keywords:** SARS-CoV-2 antibodies, seroepidemiological study, seroprevalence, National and Kapodistrian University of Athens

## Abstract

Due to early implementation of public health measures, Greece had low number of SARS-CoV-2 infections and COVID-19 severe incidents in hospitalized patients. The National and Kapodistrian University of Athens (ΝΚUA), especially its health-care/medical personnel, has been actively involved in the first line of state responses to COVID-19. To estimate the prevalence of antibodies (Igs) against SARS-CoV-2 among NKUA members, we designed a five consecutive monthly serosurvey among randomly selected NKUA consenting volunteers. Here, we present the results from the first 2500 plasma samples collected during June–July 2020. Twenty-five donors were tested positive for anti-SARS-CoV-2 Igs; thus, the overall seroprevalence was 1.00%. The weighted overall seroprevalence was 0.93% (95% CI: 0.27, 2.09) and varied between males [1.05% (95% CI: 0.18, 2.92)] and females [0.84% (95% CI: 0.13, 2.49)], age-groups and different categories (higher in participants from the School of Health Sciences and in scientific affiliates/faculty members/laboratory assistants), but no statistical differences were detected. Although focused on the specific population of NKUA members, our study shows that the prevalence of anti-SARS-CoV-2 Igs for the period June–July 2020 remained low and provides knowledge of public health importance for the NKUA members. Given that approximately one in three infections was asymptomatic, continuous monitoring of the progression of the pandemic by assessing Ig seroprevalence is needed.

## 1. Introduction

The worldwide outbreak of Severe Acute Respiratory Syndrome Coronavirus 2 (SARS-CoV-2) which emerged in Wuhan, China in December 2019 rapidly spread to more than 200 countries and as of 21 August 2020, over 22 million confirmed cases and nearly 800,000 deaths from the acute respiratory disease named coronavirus infectious disease 2019 (COVID-19) were recorded [1]. Due to the ease of viral transmission primarily through person-to-person contact, crowded and confined indoor spaces or indoor occurring events have been directly associated with higher infection rates [2]. The risk of transmission from symptomatic and pre-symptomatic patients is in general considered to be higher. However, asymptomatic infection frequently occurs and the potential of viral transmission from asymptomatic individuals bearing similar viral loads in their respiratory and gastrointestinal samples with symptomatic cases has also been reported [3,4,5].

Molecular techniques based on RT-qPCR are used to diagnose acute SARS-CoV-2 infection in principle in nasopharyngeal swabs and recovery from infection in most cases correlates with PCR negativity [6]. Within 2–3 weeks after the onset of symptoms, the immune system of infected individuals reacts by producing antibodies (Igs) to SARS-CoV-2 proteins, which can be detected in peripheral blood. As already established for other viral infections, determining anti-SARS-CoV-2 Igs is important for evaluating recovery of hospitalized patients from COVID-19, but is also the method-of-choice for evaluating SARS-CoV-2 seroprevalence in a given population [7]. Since the emergence of the pandemic, numerous serology tests were developed and marketed, although only some of them pursued Food and Drug Administration (FDA) approval. The performance of many serology tests, as defined by their sensitivity (true positive rate) and specificity (true negative rate), has been overestimated, as they were validated using samples from hospitalized, often critically ill COVID-19 patients with high anti-SARS-CoV-2 Ig titers [8]. Thus, to accurately evaluate disease epidemiology, highly sensitive and specific tests which determine SARS-CoV-2 serostatus in outpatients, asymptomatic individuals and contact persons must be preferentially applied.

Due to the early nationwide lock-down and additional public health measures, Greece was among the European countries less affected by the SARS-CoV-2 pandemic, with nearly 8000 confirmed cases and 238 deaths [1]. As of April 2020, the estimated prevalence of anti-SARS-CoV-2 Igs in the general population was 0.23% [9], although higher percentages were sporadically recorded in overpopulated or SARS-CoV-2-exposed niches, due to differences in infection rates [10].

The National and Kapodistrian University of Athens (NKUA) is amongst the biggest educational-research institutes in the larger area of the Balkans and Eastern Mediterranean basin and the most highly populated Institution in Greece, with more than 70,000 students (undergraduates and postgraduates) and over 4000 personnel (faculty members/laboratory assistants, scientific affiliates and administrative officers). Due to the structure of its curriculum, students and personnel are often placed in indoor lecture halls, laboratories and clinics, thus increasing the probability of SARS-CoV-2 transmission. Moreover, because of the significant number of its COVID-19-dedicated medical clinics, a significant proportion of NKUA personnel, especially health-care workers, had a high exposure risk. As with all educational Institutions in Greece, NKUA complied with the decision of the Greek Government and suspended its operation since 10 March 2020. To ensure safe reopening of NKUA institutional facilities, we pursued to assess SARS-CoV-2 seroprevalence among NKUA members over a period of five consecutive months (June–October 2020) in order to estimate key epidemiological parameters, namely the epidemic growth rate and the fraction of subclinical/asymptomatic infections, as well as the proportion of NKUA members who may remain susceptible to SARS-CoV-2 infection. Following analysis of the first plasma samples from 2500 NKUA members collected in June–July 2020, we present herein the results, which may eventually assist the management of the available NKUA human resources.

## 2. Materials and Methods

The study was conducted at selected NKUA locations in Athens, Greece during June–July 2020 and samples were collected from NKUA members, comprising faculty members/laboratory assistants, scientific affiliates, administrative officers, undergraduate and postgraduate students. The protocol was approved by the Ethics and Bioethics Committee of the School of Medicine, NKUA (protocol # 312/02-06-2020) and all volunteers agreed to participate in the study via signing a written inform consent.

Peripheral blood was collected by venipuncture in BD vacutainers with spray-coated K2EDTA (BD Biosciences, San Jose, CA, USA) and centrifuged at 500× *g* for 20 min. Blood plasma was transferred in DNA-RNA free cryovials (Corning, NY, USA) and frozen at −20 °C until Ig measurement, performed no later than 20 days post blood collection. Plasma samples were analyzed using the CE-IVD Roche Cobas Elecsys^®^ Anti-SARS-CoV-2, an electrochemiluminescence immunoassay (ECLIA) for the qualitative detection of total Igs (IgG, IgM and IgA; pan-Ig) generated against SARS-CoV-2 (Roche Diagnostics, Indianapolis, IN, USA). The test was performed according to the manufacturer’s instructions. The assay uses the recombinant nucleocapsid (N) protein of SARS-CoV-2 as antigen and the method is based on the well-established “double-antigen sandwich” format between donors’ plasma, biotinylated SARS-CoV-2-specific N antigen and SARS-CoV-2-specific recombinant N antigen labeled with a ruthenium complex. After concomitant incubation of reagents, streptavidin-coated microparticles are added; the immune-complex binds to the solid phase via a biotin-streptavidin interaction and is further aspirated into the measuring cell of a Roche Cobas E411 Analyzer, where microparticles are magnetically captured onto the electrode surface. Unbound material is washed out and the emission of chemiluminescence induced by the specific electrical current at the electrode is measured by a photomultiplier. Test results are generated by interpolating the ECLIA signal with that of a threshold generated during calibration. A cut-off index (COI) of 1.0 or higher classifies a plasma sample as “reactive” (i.e., anti-SARS-CoV-2 positive). The total procedure requires 20 μL of plasma and the duration of the assay is 18 min. According to the manufacturer’s package insert, Elecsys^®^ Anti-SARS-CoV-2 exhibits high overall clinical specificity of 99.81% with no cross-reactivity to the common cold coronaviruses; clinical sensitivity, determined by testing a total of 204 samples from 69 symptomatic patients with a PCR-confirmed SARS-CoV-2 infection, is 100% for samples collected ≥14 days after PCR confirmation in this collective; these values were verified in our study by measuring 25 RT-qPCR SARS-CoV-2 positive and 25 negative samples.

The prevalence of antibodies against SARS-CoV-2 was initially computed by calculating the unweighted proportions of positive tests (unweighted prevalence). Then, the final estimation of the prevalence was obtained by following a two-step approach: firstly, the unweighted proportions of positive tests was adjusted for the sensitivity and specificity of the test according to the manufacturer’s specification, as implemented in the epiR package (R version 3.6.3, R Foundation for Statistical Computing, Vienna, Austria); and secondly, the prevalence was estimated after weighting for the age distribution (18–74 years old) of the population in the Attica region (data from the 2011 census).

## 3. Results

Prior to the analysis of the NKUA donors’ samples, plasma from 50 individuals was analyzed in exactly the same way using the Roche Cobas Elecsys Anti-SARS-CoV-2^®^. Of these, 25 were archival samples, collected and kept frozen at −80 °C prior to the SARS-CoV-2 pandemic (between April–July 2019) and thus, expected to be negative; indeed, they were all classified as negative with the Roche Cobas Elecsys Anti-SARS-CoV-2^®^ (COI < 1.0). The remaining 25 plasma samples were collected from convalescence plasma donors and were prior tested for anti-SARS-CoV-2 IgGs with the commercially available EuroImmun Anti-SARS-CoV-2 enzyme-linked immunosorbent assay (ELISA; EuroImmun AG, Luebeck, Germany); 23/25 were classified as positive with the Roche assay (COI ≥ 1.0). Two samples tested negative with the Roche assay, were also negative with the EuroImmun ELISA, suggesting that the two SARS-CoV-2-infected individuals likely did not develop a measurable (by the two assays) humoral response.

We then analyzed the first cohort of 2500 samples from NKUA donors collected during June–July 2020 using the Roche Cobas Elecsys Anti-SARS-CoV-2^®^. Demographic characteristics and distribution of the participants into categories depending on occupation are shown in Table 1. A total of 25 cases were tested positive for antibodies against SARS-CoV-2; thus, the unweighted seroprevalence of anti-SARS-CoV-2 Igs was 1.00%. After adjusting for age and the indicated sensitivity (100%) and specificity (99.81%) of the test, the weighted overall seroprevalence was 0.93% (95% CI: 0.27, 2.09) (Table 2). No significant difference was observed between the weighted seroprevalence in males [1.05% (95%CI: 0.18, 2.92)] and females [0.84% (95%CI: 0.13, 2.49)]. The weighted seroprevalence for different group categories according to gender, age, whether affiliated with the School of Health Sciences and with non-health sciences departments, and position at the NKUA are shown in detail in Table 2. The weighted seroprevalence for the School of Health Sciences [1.43% (95% CI: 0.06, 6.52)] was higher than for non-health sciences departments [0.65% (95% CI: 0.04, 2.68)]; yet, this difference was not statistically significant. Differences were also observed among the personnel, with students and administrative officers having lower prevalence [0.42% (95% CI: 0.03, 1.50) and 0.48% (95%CI: 0.00, 2.37), respectively] than scientific affiliates [1.42% (95% CI: 0.00, 7.24)] and faculty members/laboratory assistants [1.20% (95% CI: 0.14, 3.70)].

In addition, a non-significant increasing trend was observed for the weighted prevalence with age (*p* = 0.157). Our statistical analysis revealed no significant associations between seroprevalence and the selected parameters (age, gender, school, position). In more detail, 14/25 (56%) of seropositive cases reported travelling mostly to European Union (EU) countries during the past 5–8 months. Similarly, 14 seropositive donors (56%) reported known contact with suspected COVID-19 cases. Among the seropositive NKUA members, only 6/25 cases (24%) stated RT-qPCR-confirmed SARS-CoV-2 infection. The most common SARS-CoV-2-related symptoms were fever and headache, whereas 12/25 (48%) individuals reported smell and/or taste loss. Finally, 9/25 (36%) of SARS-CoV-2-infected individuals were asymptomatic.

## 4. Discussion

Emerging seroprevalence studies are currently implemented worldwide as they are considered a valuable tool to reveal the extent of SARS-CoV-2 infection via estimating the proportion of the population exposed to the virus. As of now, serological surveys reported that viral spread ranges from <1.0% in countries less affected by the pandemic (e.g., Germany, Greece, Norway, Iceland) to higher levels in countries that did not implement early public health measures (5.5% in Spain, 6.0% in Belgium, 8.5% in the UK). In some heavily affected areas in Italy, Brazil and some states in the USA, seroprevalence was found to be as high as 25% [9,10,11,12,13,14,15]. Nevertheless, it was reportedly suggested that SARS-CoV-2 infections are potentially underdiagnosed and asymptomatic cases account for up to 41% [3] and in specific populations up to 59% [16] of infections.

In our preliminary results, the weighted overall seroprevalence of Igs against SARS-CoV-2 was 0.93% (95% CI: 0.27, 2.09) among 2500 donors from the NKUA sampled in June–July 2020. Our estimate is similar to the seroprevalence (0.85%) of Igs against SARS-CoV-2 found in large urban areas in Greece in a leftover IgG serosurvey using samples tested during March and April 2020 [9]. We also showed that seroprevalence varied between gender (higher in males), age groups (higher in the 55–74 years age group) and position at the NKUA (higher in faculty members/laboratory assistants), although the differences recorded were not statistically significant. As for occupation, higher prevalence was estimated in affiliates of the School of Health Sciences (1.43%) and this is in agreement with the anti-SARS-CoV-2 prevalence (1.07%) among health-care workers in two hospitals in Athens, as tested between 13 April and 15 May 2020 [10]. Although the study population and sampling periods were not identical, all previous analyses including ours, suggest that the seroprevalence of anti-SARS-CoV-2 is low in Greece. The current study included students and personnel of the largest academic Institution in the Attica region and to our knowledge is the first study in Greece comprising samples collected until end of July 2020. We should note that flight bans were lifted together with the opening of land borders at the beginning of July. Therefore, it is of importance to investigate the impact of tourism and increased mobility to the populations’ exposure to SARS-CoV-2 in the forthcoming months.

Our study provided an estimate of the exposure of the members of the NKUA to SARS-CoV-2; this knowledge is useful for the design of public health measures for the upcoming semester. Our findings that the overall seroprevalence is low and that there is no difference among members of different schools or age groups, suggest that that there is no need for tailored measures among the members of the NKUA. This knowledge is of public health importance.

The low seroprevalence found in our study was due to the early implementation of public health measures in Greece, including the lock-down and the postponement of all in person educational activities in academic Institutions, including the NKUA. Noteworthy, a significant number of NKUA volunteers work in or are in close contact with NKUA hospitals, clinics and laboratories, being at higher risk of contracting COVID-19. Indeed, affiliates of the School of Health Sciences showed the highest prevalence (1.43%) among tested groups; however, this difference was not statistically significant, thus suggesting the low levels of SARS-CoV-2 infection among health-care workers and affiliates. Moreover, a substantial number of positively tested donors (>50%) were undergraduate or postgraduate students reporting recent mobility mainly to and from EU countries, probably as part of the exchange educational visits between higher education European Universities and Institutions.

As expected in countries where the burden of COVID-19 cases was low and therefore SARS-CoV-2 prevalence is less than 1%, high false positive rates are to be encountered with antibody tests. A suggested practice to overcome this limitation is to verify seropositive results with a different immunoassay [7,11,15] and this is eventually planned, upon collection of the 5000 NKUA donors’ samples. Moreover, in the elegant study of Gudbjartsson et al. [15], pan-Ig assays instead of separately measuring IgGs, IgMs or IgAs, were shown to more accurately detect Ig levels in qPCR-positive cases, with no decrease even at four months after diagnosis. The test we used was a pan-Ig assay, as we also considered that not all potentially infected donors mount the same type, qualitatively and/or quantitatively, of humoral immune response. Indeed, post-infection kinetics of seroconversion as well as persistence of anti-SARS-CoV-2 Igs in the peripheral blood of non-hospitalized COVID-19 patients was shown to vary between individuals [17,18]. However, seronegativity in cases of symptomatic SARS-CoV-2-infected individuals who, for yet unclear reasons, develop low or even undetectable Ig titers, as well as the possibility of low antigenic stimulation in mildly infected or asymptomatic people, should be utterly considered [19]. Our analysis was based on a non-representative sample and this is a limitation of our study.

Nevertheless, the results presented herein in conjunction with available results from national or regional seroepidemiological studies in other countries, suggest that seroprevalence rates are far too low to reach protective (herd) immunity, which as estimated requires the presence of antibodies against SARS-CoV-2 in approximately 60% of the population [19,20].

## 5. Conclusions

Our preliminary report on 2500 NKUA volunteered members clearly reveals low seroprevalence of detectable Igs to SARS-CoV-2 during June–July 2020, in accordance with the low burden of SARS-CoV-2 in Greece, as compared to other EU and non-EU countries. Although limited to NKUA members, to our knowledge this is the first serosurvey using samples collected until the end of July 2020, i.e., after the lifting of travel restrictions in Greece and the EU. Our results further suggest that broader testing for the presence of anti-SARS-CoV-2 Igs is necessary to define the extent of non-recorded SARS-CoV-2-infected cases. They also highlight that NKUA members should comply with public health measures, including maintenance of social distancing and avoidance of indoor crowding.

## Figures and Tables

**Table 1:** Study population and socio-demographic characteristics.

Characteristic	Number of Participants	Percentage (%)
**Overall**	2500	100.0
**Gender**		
Male	866	34.6
Female	1599	64.0
Unknown	35	1.4
**Age group** (**in years**)		
(18–34)	1268	50.7
(35–54)	916	36.6
(55–74)	309	12.4
Unknown	7	0.3
**School**		
Health Sciences	506	20.2
Non-Health Sciences	1173	46.9
Unknown	821	32.9
**Position at the NKUA**		
Undergraduate/Postgraduate Students	1395	55.8
Scientific Affiliates	250	10.0
Faculty Members/Laboratory Assistants	312	12.5
Administrative Officers	504	20.2
Unknown	39	1.5

**Table 2:** Weighted prevalence for age and test performance of anti-SARS CoV-2.

Characteristic	Number of Anti-SARS CoV-2 Positive Participants	Unweighted Prevalence (%)	Weighted Prevalence (%) (95% Conf. Interval)
**Overall**	25	1.00	0.93 (0.27, 2.09) *
**Gender**			
Male	8	0.92	1.05 (0.18, 2.92)
Female	17	1.06	0.84 (0.13, 2.49)
Unknown	0	-	-
**Age group** (**in years**)			
(18–34)	11	0.87	0.67 (0.23, 1.35)
(35–54)	9	0.98	0.78 (0.25, 1.66)
(55–74)	5	1.62	1.42 (0.33, 3.54)
Unknown	0	-	-
**School**			
Health Sciences	3	0.59	1.43 (0.06, 6.52)
Non-Health Sciences	10	0.85	0.65 (0.04, 2.68)
Unknown	12	-	-
**Position at the NKUA**			
Undergraduate/Postgraduate Students	10	0.72	0.42 (0.03, 1.50)
Scientific Affiliates	1	0.40	1.42 (0.00, 7.24)
Faculty Members/Laboratory Assistants	6	1.92	1.20 (0.14, 3.70)
Administrative Officers	4	0.79	0.48 (0.00, 2.37)
Unknown	4	-	-

* Missing values for age: 7 out of 2500 participants (N = 2493).

## References

[1] Johns Hopkins University of Medicine, Coronavirus Resource Center. https://coronavirus.jhu.edu/map.html.

[2] Qian H., Miao T., Liu L., Zheng X., Luo D., Li Y. (2020). Indoor transmission of SARS-CoV-2. medRxiv.

[3] Byambasuren O., Cardona M., Bell K., Clark J., McLaws M.L., Glasziou P. (2020). Estimating the extent of true asymptomatic COVID-19 and its potential for community transmission: Systematic review and meta-analysis. medRxiv.

[4] Pan X., Chen D., Xia Y., Wu X., Li T., Ou X., Zhou L., Liu J. (2020). Asymptomatic cases in a family cluster with SARS-CoV-2 infection. Lancet Infect. Dis..

[5] Huang L., Zhang X., Zhang X., Wei Z., Zhang L., Xu J., Liang P., Xu Y., Zhang C., Xu A. (2020). Rapid asymptomatic transmission of COVID-19 during the incubation period demonstrating strong infectivity in a cluster of youngsters aged 16-23 years outside Wuhan and characteristics of young patients with COVID-19: A prospective contact-tracing study. J. Infect..

[6] Liu Y., Yan L.M., Wan L., Xiang T.X., Le A., Liu J.M., Peiris M., Poon L.L.M., Zhang W. (2020). Viral dynamics in mild and severe cases of CIVID-19. Lancet Infect. Dis..

[7] Pollan M., Pérez-Gómez B., Pastor-Barriuso R., Oteo J., Hernan M.A., Perez-Olmeda M., Sanmartin J.L., Fernandez-Garcia A., Cruz I., Fernandez de Larrea N. (2020). Prevalence of SARS-CoV-2 in Spain (ENE-COVID): A nationwide, population-based seroepidemiological study. Lancet.

[8] Long Q.X., Liu B.Z., Deng H.J., Wu G.C., Deng K., Chen Y.K., Liao P., Qiu J.F., Lin Y., Cai X.F. (2020). Antibody responses to SARS-CoV-2 in patients with COVID-19. Nat. Med..

[9] Bogogiannidou Z., Vontas A., Dadouli K., Kyritsi M.A., Soteriades S., Nikoulis D.J., Mouchtouri V.A., Koureas M., Kazakos E.I., Spanos E.G. (2020). Repeated leftover serosurvey of SARS-CoV-2 IgG antibodies, Greece, March and April 2020. Eurosurveillance.

[10] Psichogiou M., Karabinis A., Pavlopoulou I.D., Basoulis D., Petsios K., Roussos S., Patrikaki M., Jahaj E., Protopapas K., Leontis K. (2020). Antibodies against SARS-CoV-2 among health care workers in a country with low burden of COVID-19. medRxiv.

[11] Fisher B., Knabbe C., Vollmer T. (2020). SARS-CoV-2 IgG seroprevalence in blood donors located in three different federal states, Germany, March to June 2020. Eurosurveillance.

[12] Percivalle E., Cambiè G., Cassaniti I., Nepita E.V., Maserati R., Ferrari A., Di Martino R., Isernia P., Mojoli F., Bruno R. (2020). Prevalence of SARS-CoV-2 specific neutralising antibodies in blood donors from the Lodi Red Zone in Lombardy, Italy, as at 06 April 2020. Eurosurveillance.

[13] Hallal P., Hartwig F., Horta B., Victora G.D., Silveira M., Struchiner C., Vidaletti L.P., Neumann N., Pellanda L.C., Dellagostin O.A. (2020). Remarkable variability in SARS-CoV-2 antibodies across Brazilian regions: Nationwide serological household survey in 27 states. medRxiv.

[14] Havers F.P., Reed C., Lim T., Montgomery J.M., Klena J.D., Hall A.J., Fry A.M., Cannon D.L., Chiang C.F., Gibbons A. (2020). Seroprevalence of Antibodies to SARS-CoV-2 in 10 Sites in the United States, March 23-May 12, 2020. JAMA Intern. Med..

[15] Gudbjartsson D.F., Norddahl C.L., Melsted P., Gunnarsdottir K., Holm H., Eythorsson E., Arnthorsson A.O., Helgason D., Bjarnadottir K., Ingvarsson R.F. (2020). Humoral immune response to SARS-CoV-2 in Iceland. N. Engl. J. Med..

[16] Zhang H.J., Su Y.Y., Xu S.L., Chen G.Q., Li C.C., Jiang R.J., Liu R.H., Ge S.X., Zhang J., Xia N.S. (2020). Asymptomatic and symptomatic SARS-CoV-2 infections in close contacts of COVID-19 patients: A seroepidimiological study. Clin. Infect. Dis..

[17] Agarwal V., Venkatakrishnan A.J., Puranik A., Kirkup C., Lopez-Marquez A., Challener D.W., O Horo J.C., Binnicker M.J., Kremers W.K., Faubion W.A. (2020). Long-term SARS-CoV-2 RNA shedding and its temporal association to IgG seropositivity. medRxiv.

[18] Huang A.T., Garcia-Carreras B., Hitchings M.D.T., Yang B., Katzelnick L.C., Rattigan S.M., Borgert B.A., Moreno C., Solomon B.D., Rodriguez-Barraquer I. (2020). A systematic review of antibody mediated immunity to coronaviruses: Antibody kinetics, correlates of protection, and association of antibody responses with severity of disease. medRxiv.

[19] Altmann D.M., Douek D.C., Boyton R.J. (2020). What policy makers need to know about COVID-19 protective immunity. Lancet.

[20] Britton T., Ball F., Trapman P. (2020). A mathematical model reveals the influence of population heterogeneity on herd immunity to SARS-CoV-2. Science.

